# Profil clinique et paraclinique de la myasthénie auto-immune à Ouagadougou au Burkina Faso

**DOI:** 10.48327/mtsi.2021.169

**Published:** 2021-11-11

**Authors:** Djingri Labodi LOMPO, Nagaonlé Éric SOME, Adja Mariam OUEDRAOGO, Rodrigue P. YONLI, Ousséini DIALLO, Christian NAPON, Athanase MILLOGO, Jean KABORE

**Affiliations:** 1 CHU de Tingandogo, Unité de formation et de recherches des sciences de la santé, Université Joseph Ki-Zerbo, Ouagadougou, Burkina Faso; 2 CHU Yalgado Ouédraogo de Ouagadougou, Unité de formation et de recherches des sciences de la santé, Université Joseph Ki-Zerbo, Ouagadougou, Burkina Faso; 3 Institut de recherche en sciences de la santé Ouagadougou, Département de biologie médicale et santé publique, Ouagadougou, Burkina Faso; 4 CHU Souro Sanou de Bobo-Dioulasso, Unité de formation et de recherches des sciences de la santé, Université Joseph Ki-Zerbo, Ouagadougou, Burkina Faso

**Keywords:** Myasthénie auto-immune, Classe mgFA IV/V, Ac anti-RACh, Ac anti-MuSK, Thymome, Hôpital, Ouagadougou, Burkina Faso, Afrique subsaharienne, Autoimmune myasthenia gravis, mgFA classe IV/V, Anti-RACh Ab, Anti-MuSK Ab, Thymoma, Hospital, Ouagadougou, Burkina Faso, Sub-Saharan Africa

## Abstract

**Introduction:**

Certaines différences de profils épidémiologique, clinique, paraclinique et évolutif de la myasthénie auto-immune (MAI) sont de plus en plus décrites chez les patients selon les origines géographiques. La présente étude a été réalisée afin de caractériser le profil sociodémographique, clinique et paraclinique de la MAI à Ouagadougou, au Burkina Faso.

**Patients et méthodes:**

C'est une étude transversale, descriptive, réalisée dans les structures de santé de Ouagadougou (Burkina Faso), sur une période de 5 ans 6 mois, de mars 2015 à septembre 2019. L’étude a concerné tous les patients qui avaient une symptomatologie clinique évocatrice de myasthénie, associée à la présence dans le sérum d'anticorps (Ac) anti-RACh et/ou d'Ac anti-MuSK et/ou la présence d'un décrément >10% en électroneuromyographie et/ou un test thérapeutique positif aux anticholinesthérasiques oraux. Les données sociodémographiques, cliniques et paracliniques, ont été analysées.

**Résultats:**

En tout 25 patients, soit 15 femmes (60%) et 10 hommes (40%), ont été colligés. La forme de l'adulte jeune avec 20 cas (80%) était prédominante. Le délai médian entre les premiers symptômes et le diagnostic était de 26 mois (2 - 217 mois). Une diplopie et/ou un ptosis (80%) et une dysphonie (72%), étaient les présentations cliniques révélatrices les plus fréquentes. À l'admission, 7 patients (28%) avaient une forme généralisée modérée (classe de la *Myasthenia Gravis Foundation of America* (MGFA) III) et 9 patients (36%) avaient une forme généralisée sévère à très sévère (classe mgFA IV à V). Les dosages plasmatiques des Ac ont été réalisés chez 17 patients (68%): les Ac anti-RACh étaient positifs chez 11 patients (64,7%) et les Ac anti-MuSK chez 3 patients (14,3%). La TDM thoracique a objectivé une hyperplasie du thymus chez 12 patients (48%), un thymome chez 5 patients (20%). Une hyperthyroïdie était observée chez 2 patients (8%).

**Conclusion:**

La MAI à Ouagadougou, au Burkina Faso est marquée par un retard diagnostic, une prédominance chez la femme jeune, des formes généralisées sévères et une fréquence élevée des Ac anti-MuSK plasmatiques. Ce profil semble différent de celui des patients d'origine caucasienne. Des études collaboratives dans la région subsaharienne sur la MAI en populations générales sont nécessaires.

## Abstract (en)

**Introduction:**

Certain differences in the epidemiological, clinical, paraclinical and evolution profiles of autoimmune myasthenia gravis (AIMG) are increasingly described in patients according to geographic origins. The present study was carried out in order to help characterize the socio-demographic, clinical and paraclinical profile of AIMG in Ouagadougou, Burkina Faso.

**Patients and methods:**

This was a cross-sectional, descriptive, multicenter, hospital study carried out in Ouagadougou (Burkina Faso), over a period of 5 years 6 months, from March 2015 to September 2019. The study concerned all patients who had a clinical symptomatology suggestive of myasthenia gravis, associated with the presence in the serum of anti-AChR Ab and / or anti-MuSK Ab and / or the presence of a >10% decrement in electroneuromyography and / or a positive therapeutic test for oral anticholinestherasics. Sociodemographic, clinical and paraclinical variables, were analyzed.

**Results:**

A total of 25 patients (15 women and 10 men), were included. The young adult form was predominant (20 cases). The median time between the first symptoms and the diagnosis was 28.4 months +/- 44.8 (2 - 217 months). Diplopia and/or ptosis (80%) and dysphonia (72%) were the most frequent revealing clinical presentations. On admission, 7 patients (28%) had a moderate generalized form (MGFA class III) and 9 patients (36%) had a severe to very severe generalized form (MGFA class IV to V). Plasma Ab assays were performed in 17 patients (68%): anti-RACh Ab were positive in 11 patients (64.7%) and anti-MuSK Ab in 3 patients (14.3%). Thoracic CT revealed thymus hyperplasia in 12 patients (48%), thymoma in 5 patients (20%). Hyperthyroidism was associated in 2 patients (8%).

**Conclusion:**

AIMG in Ouagadougou, Burkina Faso is marked by delayed diagnosis, a predominance in young women, severe generalized forms and a high frequency of plasma anti-MuSK Ab. This profile appears to be different from that of Caucasian patients. Collaborative studies in the sub-Saharan region on AIMG in general populations are needed.

## Introduction

La myasthénie (myasthenia gravis) est une affection auto-immune de la jonction neuromusculaire caractérisée par une fatigabilité et une faiblesse musculaires s'aggravant à l'effort et cédant au repos, due à la présence d'anticorps spécifiques dirigés contre les protéines de la membrane post synaptique de la jonction neuromusculaire: récepteur nicotinique de l'acétylcholine (RACh), muscle spécifique kinase (MuSK), low-density lipoprotein receptor-related protein 4 (LRP4) (protéine 4 liée aux récepteurs des lipoprotéines de basse densité), agrine, titine, ryanodine. Les patients atteints de MAI sont actuellement classés en sous-types basés sur la présentation clinique et les Ac sériques: MAI à début précoce avec Ac anti-RACh (âge de début des premiers symptômes avant 50 ans), MAI à début tardif (âge de début des premiers symptômes après 50 ans), MAI avec Ac anti-RACh, MAI associée au thymome, MAI avec Ac anti-MuSK, MAI avec Ac anti-LRP4, MAI généralisée séronégatives et MAI oculaire [9, 13].

Son incidence globale moyenne est de 5,3 par million de personnes-années [9]. Selon la situation géographique, la prévalence de la MAI varie entre 1,5 et 17,9 cas/100 000 habitants dans les populations à majorité occidentale [10] ou de 2,19 à 36,7 cas/100 000 habitants à travers le monde [1, 9], avec une tendance à l'augmentation régulière de la prévalence ces dernières décennies, imputable à une amélioration de la survie des patients, à l'amélioration des moyens diagnostics et au vieillissement de la population [9].

La MAI affecte tous les âges, mais elle est plus fréquente chez les jeunes femmes et les personnes âgées. Il existe une prédominance féminine de la maladie avant l’âge de 40 ans, qui s'atténue par la suite jusqu’à l’âge de 50 ans; au-delà de 60 ans, les hommes sont les plus atteints par la maladie [1]. L’âge d'apparition le plus fréquent se situe entre 20 et 39 ans chez la femme [5] et entre 50 et 70 ans chez l'homme [20]. Une augmentation constante de l'incidence et de la prévalence de la MAI à début tardif a été constatée dans les pays occidentaux [27] et asiatiques [25], ces 3 dernières décennies, en raison d'un probable contexte immunologique particulier, notamment l'augmentation de la tolérance aux maladies auto-immunes avec l’âge et/ou de facteurs environnementaux, notamment la prolongation de l'espérance de vie dans ces régions [9].

La MAI juvénile, un sous-type de maladie à début précoce, a une fréquence élevée en Asie de l'Est (Chine, Taiwan, Japon), où jusqu’à 50% de tous les cas apparaissent avant l’âge de 15 ans, beaucoup d'entre eux avec symptômes oculaires uniquement [10, 35], alors que ce sous-type est plus rare en Europe et en Amérique du Nord où il ne représente que 10 à 15% des malades [35].

En Afrique subsaharienne (ASS), la MAI est caractérisée par de longs délais diagnostics, atteignant en moyenne 24 mois au Sénégal [34], des difficultés d'accès aux outils diagnostics, une plus grande fréquence des formes graves [21]. Certaines études comparatives aux USA [28] et en Afrique du Sud [17], et certaines revues systématiques [9, 18], ont rapporté une fréquence plus élevée des formes à début précoce, une prédominance plus marquée du genre féminin, une plus faible fréquence des Ac anti-RACh, une fréquence plus élevée des Ac anti-MUSK et une tendance à des formes plus sévères de la maladie, chez les patients d'origine noire africaine comparés à ceux principalement d'origine européenne. Ces observations nous ont conduit à réaliser la présente étude dont l'objectif était de caractériser le profil socio-démographique, clinique et paraclinique de la MAI à Ouagadougou, au Burkina Faso.

## Patients et Méthodes

C'est une étude transversale, descriptive, multicentrique, ayant concerné toutes les structures de santé privée et publique de Ouagadougou, qui a couvert une période de 5 ans 6 mois, allant de mars 2015 à septembre 2019.

Avant le début de l’étude, une correspondance officielle avait été transmise à tous les médecins exerçant dans les différentes structures sanitaires publiques comme privées et aux différents responsables administratifs desdites structures sanitaires afin qu'ils nous réfèrent tout cas suspect de myasthénie (patients présentant une symptomatologie clinique évocatrice de myasthénie) ou tout cas de myasthénie déjà confirmée. Sur un total de 368 structures de santé comptabilisées dans la ville de Ouagadougou (7 hôpitaux, 11 centres médicaux urbains et 350 cliniques et cabinets médicaux privés), nous en avons sollicité 218 (7 hôpitaux, 11 centres médicaux urbains et 200 structures de santé privées), parmi lesquelles 199 ont collaboré et 36 structures nous ont référé des cas suspects. Les cas confirmés provenaient de 4 hôpitaux, 2 centres médicaux urbains et 3 cliniques privées.

Ainsi les patients qui nous ont été référés ont été régulièrement reçus en consultation externe de neurologie dans les 3 principaux CHU de la ville de Ouagadougou (Burkina Faso): CHU Tingandogo (CHU-T), CHU Yalgado Ouédraogo et CHU de Bogodogo. Les patients qui ne pouvaient faire le déplacement ont été vus dans la structure sanitaire de base, après déplacement express du neurologue.

Ont été inclus dans notre étude tous les patients qui avaient une symptomatologie clinique évocatrice de myasthénie, associée à au moins un des critères suivants: présence dans le sérum d'Ac anti-RACh à un taux > 0,4 nmol/ml (négatif ou douteux si ≤ 0,4 nmol/ml) et/ou présence d'Ac anti-MuSK; présence d'un décrément >10%, au minimum sur 2 couples nerf-muscle, lors des stimulations répétitives à 3 cycles/seconde en électroneuromyographie, après arrêt des anticholinestérasiques la veille de l'examen; test thérapeutique par les anticholinesthérasiques oraux [pyridostigmine bromide, comprimés 60 mg (Mestinon^®^) ou ambenonium chloride, comprimés 10 mg (Mytelase^®^)] révélant un bénéfice fonctionnel net dans la vie quotidienne du patient sur une durée d'au moins 14 jours. En tout 78 cas suspects de myasthénie ont bénéficié d'un test thérapeutique principalement au pyridostigmine bromide comprimés 60 mg per os toutes les 4 heures du lever le matin au coucher nocturne pendant au moins 14 jours.

En tout 96 patients nous ont été référés pour suspicion de myasthénie.

N'ont pas été inclus dans l’étude les patients dont la symptomatologie clinique était évocatrice de myasthénie, mais non confirmée par les examens paracliniques et/ou ne répondant pas au test thérapeutique par anticholinestérasique oral.

Les dosages des Ac anti-RACh et anti-MuSK n’étant pas réalisables sur place au Burkina Faso, tous les prélèvements de sang en vue de leurs dosages ont été pratiqués puis conditionnés au sein d'un seul laboratoire de référence à Ouagadougou (laboratoire Philadelphie) disposant d'une convention de coopération avec un laboratoire de référence en France. Par la suite, les prélèvements pratiqués par une équipe de biologistes expérimentés, ont été envoyés vers le laboratoire de référence en France, par vols réguliers. Les dosages proprement dits des Ac anti-RACh et des Ac anti-MuSK ont été réalisés au laboratoire de biologie médiale Cerba en France, selon la méthode de dosage radio-immunologique pour les Ac anti-RACh et la méthode d'enzymo-immunologie par test ELISA pour les Ac anti-MuSK.

Les examens d'ENMG ont été réalisés dans les laboratoires de neurophysiologie clinique des CHU de Tengandogo et de Bogodogo sur le même type d'appareils de marque Neurosoft^®^.

Les variables d’étude prises en compte étaient:

- sociodémographiques: âge, sexe, délai de consultation en neurologie;
- cliniques: motifs de consultation (symptômes/signes myasthéniques), antécédents médicaux, modes d'installation (aigu/subaigu/progressif), signes fonctionnels, délai diagnostic, scores musculaires moteurs myasthéniques (SMM), stades évolutifs selon la *Myasthenia Gravis Foundation of America* (MGFA);
- paracliniques: test pharmacologique au pyridostigmine bromide ou au ambenonium chloride, dosage sérique des Ac anti-RACh et anti-MuSK, résultats de la TDM thoracique (présence ou non d'une anomalie du thymus), résultats de l'ENMG, bilan sérique auto-immun, dosage sanguin des hormones thyroïdiennes, examen anatomopathologique des pièces d'exérèse des anomalies du thymus).

Le score musculaire moteur (SMM) a été utilisé pour évaluer l'importance de la fatigabilité musculaire lors de l'examen clinique initial et ultérieurement lors des différentes consultations neurologiques de suivi. C'est un score analytique quantitatif côté de 0 à 100 points. Lors de l'examen clinique initial, les patients ont été subdivisés en 5 sous-groupes de gravité clinique croissante selon la classification internationale de la mgFA [18].

L’étude a été autorisée par le Comité national d’éthique du Burkina Faso. La collecte des données a été effectuée avec l'autorisation des administrations des différentes structures sanitaires. Les fiches de collecte ont été remplies sur place après avoir obtenu le consentement des patients ou de leurs tuteurs légaux; les données recueillies sont restées confidentielles.

## Résultats

Durant la période d’étude nous avons colligé consécutivement 25 patients, soit 15 femmes (60%) et 10 hommes (40%), soit un sex-ratio F/H de 1,5.

La médiane d’âge lors du diagnostic était de 27 ans (extrêmes: 7 - 59 ans). La tranche d’âge de 20 à 40 ans lors du diagnostic, avec 17 patients (68%) était plus représentative.

La médiane d’âge de début des symptômes était de 25 ans (extrêmes: 5-56 ans). L’âge de début des symptômes se situait entre 10 et 30 ans chez la majorité des patients, soit 17 patients sur 25 (64%).

L’âge de début des symptômes se situait dans la tranche d’âge de 5 à 40 ans, chez la quasi-totalité des femmes, soit 14 patientes (93%) sur 15, versus 7 patients sur 10 (70%) pour les hommes. Pour la tranche d’âge de plus de 50 ans, seuls les hommes étaient représentés (2 patients sur 10, soit 20%).

**Figure 1:**
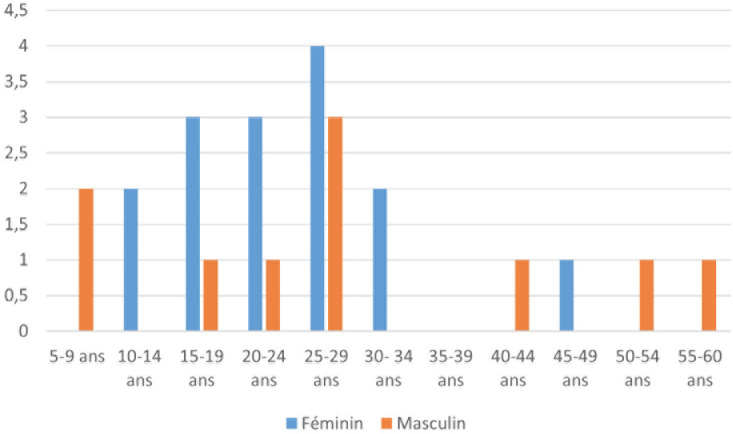
Répartition des patients selon l’âge du début des symptômes et le genre Distribution of patients by age of symptom onset and gender

Selon l’âge de début, nous avons recensé 3 cas (12%) de forme juvénile débutant avant l’âge de 15 ans, 20 cas (80%) de myasthénie de l'adulte jeune (âge de début ≤ 50 ans) et 2 cas (8%) de myasthénie à début tardif (>50 ans) (Fig. 1)

Des comorbidités médicales étaient présentes chez 7 patients (28%), lors du diagnostic. Il s'agissait de l'asthme (2 patients), séquelles d'AVC, diabète type 2, HTA, drépanocytose, hépatite virale chronique B, respectivement chez un patient chacun. Des antécédents d'intervention chirurgicale ont été retrouvés chez 9 patients (36%).

Aucune notion de cas de myasthénie familiale n'a été rapportée dans notre série. Le délai médian entre le début des symptômes et le diagnostic était de 26 mois (extrêmes 2 - 217 mois).

Le mode d'installation de la maladie était subaigu chez 6 patients (24%), et progressif chez 19 patients (76%).

Les circonstances de survenue étaient connues chez 10 patients (40%). Il s'agissait du stress et du surmenage physique chez 3 patients (12%) chacun, de traumatisme et de prise médicamenteuse chez 2 patients (8%) chacun et du post-partum chez une patient (4%).

**Figure 2:**
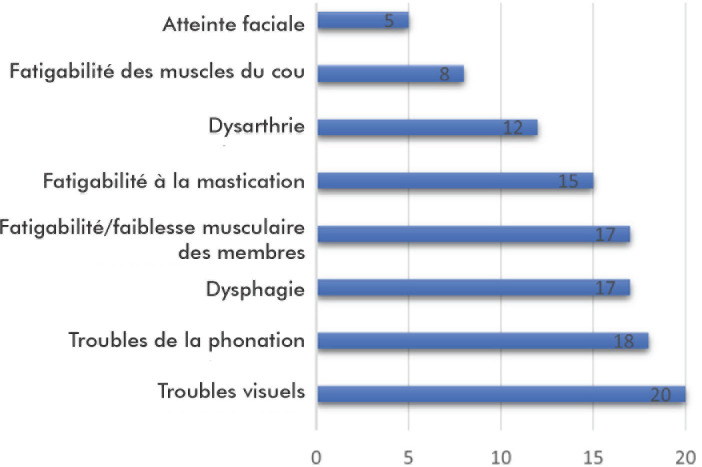
Répartition des patients selon la présentation clinique révélatrice Distribution of patients according to the revealing clinical presentation

Les troubles visuels à type de diplopie et/ou ptosis chez 20 patients (80%), la dysphonie chez 18 patients (72%), la dysphagie et la faiblesse ou fatigabilité des muscles des membres, respectivement chez 17 patients (68%) chacun, étaient les présentations cliniques révélatrices les plus fréquemment rencontrées (Fig. 2).

Lors de l'examen clinique initial, un amaigrissement chez 7 patients (28%), une amyotrophie des ceintures scapulaires et pelviennes chez 5 patients (20%) et une amyotrophie massétérine et de la langue chez 2 patientes (8%) étaient les signes cliniques retrouvés.

Lors de l'examen clinique initial, le score musculaire moteur (SMM) moyen était de 50,2 points /100 ± 10,5 (22-100 points); il y avait 22 patients / 25 (88%) avec une forme généralisée contre 3 patients (12%) qui avaient une forme oculaire. Selon la classification de la mgFA initiale à l'admission, 7 patients (28%) avaient une forme généralisée modérée (classe mgFA III) et 9 patients (36%) avaient une forme généralisée sévère à très sévère (classe mgFA IV à V) (Tableau I).

**Tableau I:** Répartition des patients myasthéniques selon la sévérité clinique initiale (lors de l'admission) Distribution of myasthenic patients according to initial clinical severity (on admission)

Sévérité clinique initiale	Effectifs (n)	%
Classe mgFA I	3	12
Classe mgFA II	6	24
Classe mgFA III	7	28
Classe mgFA IV	5	20
Classe mgFA V	4	16
**Total**	25	100

MGFA = *Myasthenia Gravis Foundation of America*

Les dosages plasmatiques des Ac anti-RACh et Ac anti-MuSK ont été réalisés chez 17 patients (68%), chacun; ce dosage n'a pas été effectué chez 8 patients (32%) pour des raisons d'inaccessibilité financière. Le dosage des Ac anti-RACh est revenu positif chez 11 patients (64,7%). Sur les 11 résultats positifs, un (9%) avait une MAI oculaire et dix (90%) une MAI généralisée. Ce dosage était négatif chez 6 patients (35%) sur l'ensemble des 17 patients qui l'ont réalisé. Le dosage des Ac anti-MuSK est revenu positif chez 3 patients (14%), dont un cas de double séropositivité des Ac anti-RACh et Ac anti-MuSK. L'ENMG de stimulation nerveuse répétitive a été effectué chez 23 patients (92%); il a objectivé un décrément significatif (> 10%) dans tous les cas (100%).

Une TDM thoracique a été réalisée chez tous nos patients. Elle a objectivé une hyperplasie du thymus chez 12 patients (48%), un thymome chez 5 patients (20%) et un aspect normal chez 8 patients (32%).

Un examen anatomo-pathologique a été réalisé pour 9 patientes (36%) après thymectomie et a permis de retrouver un thymome de type A chez 5 patients et une hyperplasie folliculaire chez 4 patients.

Le dosage des Ac anti-thyroglobuline, anti-facteur intrinsèque, anti-DNA natifs, anti-nucléaires ont été réalisés chez 9 patients (36%) et sont revenus normaux dans tous les cas. Le dosage des hormones thyroïdiennes a été réalisé chez tous nos patients (100%) et a objectivé une hyperthyroïdie chez 2 patients (8%).

Selon l’âge de début de la maladie, 3 sous-groupes de patients ont été identifiés:

- les patients du sous-groupe infanto-juvénile représentait 16% de l'effectif, avec une moyenne d’âge de 10 ans, sans prédominance de genre, ne comportait que des formes focales oculaires ou généralisées légères; les Ac anti-RACh étaient positifs chez 25% et les formes séronégatives étaient notées chez 50% des cas; la présence d'une hyperplasie thymique et d'un thymome étaient retrouvée respectivement dans 50% des cas chacun;
- les patients du sous-groupe de la MAI de l'adulte jeune représentait 76% de l'effectif, avec une moyenne d’âge de 26,7 ans, une prédominance féminine (68%), des formes généralisées exclusives (100%) avec une prédominance des formes généralisées sévères à très sévères (68%), des Ac anti-RACh positifs chez 68% et des Ac anti-MuSK positifs chez 15%, une hyperplasie thymique et un thymome retrouvés respectivement chez 47% et 21% des patients;
- les patients du sous-groupe de la MAI du sujet âgé représentait 8% de l'effectif, avec une exclusivité masculine, une moyenne d’âge de 53,5 ans, composée exclusivement de formes généralisées sévères à très sévères, des Ac anti-RACh positifs et une hyperplasie thymique présente, chez 50% des cas chacun (Tableau II).

**Tableau II:** Caractéristiques des sous-groupes de MAI selon l’âge de début Characteristics of AIMG subgroups by age of onset

Caractéristiques	MAI infanto-juvénile (1-15 ans)	MAI de l'adulte jeune (16-50 ans)	MAI du sujet âgé (>50 ans)
**Effectifs**	4 patients (16%)	19 patients (76%)	2 patients (8%)
**Moyenne d’âge**	10 ans (4-15 ans)	26,7 ans (18-46 ans)	53,5 ans (52-55 ans)
**Genre**	autant de femmes que d'hommes	prédominance féminine: 13 patientes sur 19 (68,4%)	exclusivement masculine
**SMM moyen initial**	83,7 points sur 100	44,4 points sur 100	39 points sur 100 (30-48)
**Classe mgFA initial**	I ou II: 4 cas sur 4 (100%)	II ou III: 6 sur 19 (31%) IV ou V: 13 sur 19 (68%)	IV à V: 2 cas sur 2 (100%)
**Déficit moteur persistant** aucun	7 cas sur 19 (36,8%)	1 cas sur 2
**Sérologie**	Ac anti-RACh +: 1 cas sur 4 (25%) séronégatif: 2 cas sur 4 (50%) inconnue: 1 cas sur 4 (25%)	Ac anti-RACh +: 9 cas sur 19 (47%) Ac anti-MuSK +: 3 cas/19 (15%) séronégatif: aucun Inconnue: 4 cas sur 19 (21%)	Ac anti-RACh+: 1 cas sur 2 séronégatif: 1 cas sur 2
**TDM thoracique**	hyperplasie thymique: 2 cas sur 4 (50%) thymome: 1 cas sur 4 (25%) thymus normal: 1 cas sur 4 (25%)	hyperplasie thymique: 9 sur 19 (47%) thymome: 4 sur 19 (21%) thymus normal: 6 sur 19 (31%)	hyperplasie thymique: 1 cas sur 2 thymus normal: 1 cas sur 2

SMM = score musculaire moteur; TDM = tomodensitométrie;

## Discussion

La MAI touche tous les âges, mais elle est considérée comme « une maladie des jeunes femmes et des hommes âgés » [10].

L’âge d'apparition le plus fréquent se situe entre 20 et 39 ans chez la femme [5] et entre 50 et 70 ans chez l'homme [20]. L’âge d'apparition est plus élevé chez les patients occidentaux que chez les Noirs américains [31]. En effet, l’âge d'apparition de la MAI était de 51,8 ans chez les Américains blancs versus 33,5 ans chez les Noirs américains (p<0,0001) [28]. Les séries d'ASS, comme la nôtre, avec une moyenne d’âge de début de la maladie entre 25 ans et 32 ans [29, 30, 34], confirment cette tendance à un début plus précoce de la MAI dans les populations noires. La même tendance est observée en Chine [35], Singapour [2], Trinidad et Tobago [23]. Par contre en Iran [32] et aux Pays-Bas [6], une moyenne d’âge de début après 40 ans est notée.

Une augmentation constante de la MAI à début tardif a même été observée dans les pays occidentaux [27] et asiatiques [25] au cours de ces 30 dernières années. Cette augmentation a été rapportée à une meilleure reconnaissance de la maladie, à l'amélioration des tests de diagnostic, aux particularités immunologiques des personnes âgées ou à des facteurs environnementaux spécifiques [9].

Dans les séries occidentales, la courbe d’âge d'apparition est constamment bimodale pour les femmes, avec un pic d'apparition précoce entre 20 et 39 ans, et un pic d'apparition tardive, et tend à ne présenter qu'un seul pic d'apparition tardive entre 50 et 70 ans pour les hommes [9]. Cette tendance n'a pas été entièrement observée dans notre étude, notamment l'absence de femmes dans les tranches d’âge d'apparition tardive de la maladie. Cependant, le pic d'apparition tardive de la MAI semble moins exprimé chez nos patients africains, au même titre que chez les patients japonais, indiens et chinois atteints de MAI [9, 20].

La prédominance féminine de la MAI est plus prononcée au sein des populations d'origine hispanique, asiatique et afro-américaine comparativement aux populations d'origine européenne, probablement à cause de leur prédisposition à développer des maladies auto-immunes [31]; cette tendance a été retrouvée dans notre série. Les hormones sexuelles semblent y jouer un rôle central et les œstrogènes semblent en être le médiateur principal [10, 15].

L'origine géographique peut ainsi influencer les caractéristiques sociodémographiques de la MAI, suggérant que des facteurs génétiques ou environnementaux/de mode de vie contribuent à un phénotype spécifique, mais aussi que la MAI peut être considérée comme un ensemble de symptômes associés à différentes susceptibilités génétiques plutôt qu'une entité clinique unique. La MAI surviendrait sur un terrain génétiquement déterminé (système HLA) après exposition à divers facteurs environnementaux. Le stress émotionnel, les traumatismes physiques, le péripartum, certaines substances chimiques notamment médicamenteuses, qui figurent parmi les facteurs les plus fréquemment incriminés [9, 15], ont été retrouvés dans notre étude.

Les patients atteints de MAI oculaire et d'apparition précoce présentent une fréquence accrue de maladies auto-immunes générales et spécifiques d'organe, en particulier les maladies de la thyroïde [12], tendance effectivement observée dans notre étude.

Avec un délai médian entre les premiers symptômes et le diagnostic de 26 mois dans notre étude, un délai moyen, respectivement, > 24 mois chez plus 50% des patients aux Pays-Bas [4], de 24 mois rapportés au Sénégal [34], 24 mois chez 80% des patients en Italie [24], les patients myasthéniques accusent effectivement un retard diagnostic, partout au monde, découlant d'une errance diagnostique fréquente pouvant s’étaler sur plusieurs années. En effet, la myasthénie, particulièrement pour le médecin généraliste, est mal connue du fait de sa relative rareté, du caractère fréquemment intermittent des manifestations cliniques et donc de l'absence de signes objectifs au moment de la consultation; le risque est grand de négliger des symptômes tels que la fatigue, trop vite mise sur le compte d'un stress ou d'une dépression débutante. Dans notre contexte, l'errance diagnostique est imputable au long parcours de soins de plus de 24 mois avant la consultation neurologique qui garantit le diagnostic en moins d'un mois. Par contre, certaines séries rapportent des délais diagnostics relativement courts de 5 à 6 mois [8], du fait d'une bonne médicalisation et d'un dépistage précoce de la myasthénie par les médecins.

La symptomatologie clinique initiale de la MAI, tous âges confondus, débute habituellement dans 60 à 85% des cas par une atteinte oculaire, dans 15 à 17% des cas par une atteinte de la musculature d'innervation bulbaire ou faciale et dans 10% des cas par un déficit de la musculature axiale ou périphérique [8, 15, 23]. Dans notre série, le même profil clinique révélateur a été observé, cependant à des fréquences plus élevées pour les atteintes bulbaires et des membres, liées à des biais d'imprécisions dans la chronologie de l'histoire de la maladie favorisés par les longs délais diagnostiques dans notre contexte.

Lors de l'examen clinique initial de nos patients, jusqu’à 36% d'entre eux avaient une forme généralisée sévère à très sévère (classe mgFA IV à V) avec un SMM moyen initial de 50,2 points /100. Ces données sont le reflet probable d'un diagnostic tardif aux stades de gravité maximale de la maladie pour une bonne proportion des patients, ou peuvent entrer dans le cadre de la tendance à une plus grande fréquence des formes graves de MAI déjà observée chez les noirs américains comparativement aux américains d'origine caucasienne [28].

Dans la littérature, les Ac anti-RACh sont retrouvés chez 75% à 90% des MAI généralisées, et chez 50% à 60% des MAI oculaires [9, 13, 35], alors que dans notre série, les Ac anti-RACh étaient positifs chez 64%. Ce faible taux de séropositivité aux Ac anti-RACh dans notre étude pourrait s'expliquer par le faible taux de réalisation du dosage des Ac anti-RACh, qui n’était que de 68% et la non répétition de ce dosage en cas de séronégativité lors du dosage initial, du fait de son coût onéreux. En effet, la répétition du dosage plasmatique des Ac anti-RACh permet d'augmenter son rendement diagnostic en cas de séronégativité initiale [3].

Selon certaines études, la fréquence des Ac anti-MuSK parmi les patients atteints de MAI est globalement faible dans les populations caucasiennes, de l'ordre de 4% [13]. Selon d'autres études, les Ac anti-MuSK seraient plus fréquents chez les patients d'origine noire africaine aux USA [28] et chez les patients d'origine asiatique [7]. Cette tendance semble confirmée dans notre étude.

La fréquence des thymomes associés à la MAI se situe entre 15 à 30% [22], englobant ainsi le taux de 20% rapporté dans notre série. A noter cependant, des taux plus élevés de thymomes, de 40% au Sénégal [11] et 50% au Benin et au Gabon [14].

Ces différences de fréquences pourraient s'expliquent par les différences de profils de patients myasthéniques selon les études.

Dans notre série, seuls 8% des patients, essentiellement des enfants, avaient une forme oculaire pure, alors que la plupart des études rapportent une fréquence plus élevée de MAI oculaires au sein des populations noires [26, 31]. Ainsi la MAI oculaire pure a été rapportée chez 40% des patients à Trinidad et Tobago [23], 47% des cas en Tanzanie [19] et 50% des cas au Nigéria [29], confirmant la tendance à une plus grande fréquence de la MAI oculaire chez les sujets d'origine noire africaine. De même, la MAI oculaire est également plus fréquente dans les populations asiatiques, pouvant atteindre jusqu’à 30-56% des cas, avec une nette prédominance infantile [6,9,20,27,33,35]. À l'inverse, cette forme clinique ne représente que 10 à 15% des cas dans la population caucasienne [1, 9]. La faible fréquence de la MAI oculaire pure dans notre contexte pourrait être due aux retards diagnostics aux stades généralisés de la maladie, et au fait que peu de patients consultent le neurologue du fait du caractère relativement bénin de la forme oculaire et de la non-perception par les patients, leur entourage ou leurs médecins, des symptômes oculaires de caractère fluctuant comme possiblement myasthéniques. La faible représentativité de la MAI à début tardif dans notre contexte pourrait s'expliquer par l'espérance de vie relativement faible autour de 60 ans dans notre contexte et les nombreuses comorbidités associées au vieillissement, faisant rarement suspecter la myasthénie. Des études complémentaires en population contribueront à fournir des données épidémiologiques plus fiables.

## Limites et forces de notre étude

Le faible effectif de nos patients, l'absence de dosages des Ac anti-RACh, des Ac anti-MuSK des examens d'auto-immunité plasmatiques chez une proportion relativement importante de nos patients, faute de moyens financiers, a pu induire des biais. L'absence de dosage plasmatique des Ac chez 32% des patients a ainsi pu induire des biais dans la répartition des sous-groupes sérologiques de patients myasthéniques dans notre étude. De même, le caractère hospitalier de notre étude, a pu induire un biais de sélection des cas les plus graves aux dépens des formes cliniques oculaires pures ou généralisées légères.

Ces limites sont cependant largement contrebalancées par les forces de notre étude: la réalisation des dosages plasmatiques des Ac en utilisant les approches recommandées dans le même laboratoire pour la majorité de nos patients; la combinaison des critères diagnostiques cliniques, électrophysiologiques et biologiques pour la majorité de nos patients; le caractère multicentrique de l’étude,…malgré les difficultés liées à notre contexte de pays à faible revenu.

## Conclusion

Notre étude révèle que la MAI à Ouagadougou au Burkina Faso, est une affection prédominante de la jeune femme, marquée par un retard diagnostic du fait d'une longue errance diagnostique, une fréquence élevée des formes généralisées sévères contrastant avec une faible fréquence des formes oculaires et juvéniles, un faible accès aux examens immunologiques, une fréquence élevée des Ac anti-MuSK. A l'exception de la rareté de la forme oculaire pure, notre étude confirme que le profil épidémiologique, clinique et paraclinique des patients atteints de MAI d'origine noire africaine semble différent de celui des patients d'origine caucasienne, déjà décrit dans la littérature. Ces constats suggèrent que la MAI est une entité hétérogène associée à différentes susceptibilités génétiques et environnementales. Des études complémentaires multicentriques collaboratives dans la région subsaharienne sur la MAI sur de grands effectifs en population sont nécessaires afin d'affiner ces données préliminaires.

## Contribution des auteurs

Conception de l’étude, rédaction, révision et validation du protocole, recueil des données, analyse, rédaction et correction du manuscrit: LOMPO Djingri Labodi

Rédaction, révision et validation du protocole, recueil des données, analyse: SOME Nagaolé Eric, OUEDRAOGO Adja Mariam, YONLI P Rodrigue

Validation du protocole, correction du manuscrit: DIALLO Ousséni, NAPON Christian, MILLOGO Athanase, KABORE B Jean

## Liens d'intérêt

Les auteurs ne déclarent aucun lien d'intérêt

## Source de financement

Personnelle.

## References

[1] Alkhawajah NM, Oger J (2013). Late-onset myasthenia gravis: a review when incidence in older adults keeps increasing. Muscle Nerve.

[2] AU Wing Lok, DAS ABPN Asha, TL TJIA Helen, MMed FAMS (2003). Myasthenia gravis in Singapore. Neurol J Southeast Asia.

[3] Eymard B (2009). Anticorps dans la myasthénie. Rev Neurol (Paris).

[4] Beekman R, Kuks JB, Oosterhuis HJ (1997). Myasthenia gravis: diagnosis and follow-up of 100 consecutive patients. J Neurol.

[5] Beghi E, Antozzi C, Batocchi AP, Cornelio F, Cosi V, Evoli A, Lombardi M, Mantegazza R, Monticelli ML, Piccolo G (1991). Prognosis of myasthenia gravis: a multicenter follow-up study of 844 patients. J Neurol Sci.

[6] Berrih-Aknin S, Le Panse R (2014). Myasthénie et auto-anticorps : physiopathologie des différentes entités. Rev Med Interne.

[7] Boldingh MI, Maniaol A, Brunborg C, Dekker L, Lipka A, Niks EH, Verschuuren J, Tallaksen C (2017). Prevalence and clinical aspects of immigrants with myasthenia gravis in northern Europe. Muscle Nerve.

[8] Boughammoura-Bouatay A, Chebel S, Younes-Mhenni S, Frih-Ayed M (2008). Myasthénie de révélation tardive: à propos d'une population tunisienne. NPG Neurologie-Psychiatrie-Gériatrie.

[9] Bubuioc AM, Kudebayeva A, Turuspekova S, Lisnic V, Leone MA (2021). The epidemiology of myasthenia gravis. J Med Life.

[10] Carr AS, Cardwell CR, McCarron PO, McConville J (2010). A systematic review of population based epidemiological studies in Myasthenia Gravis. BMC Neurol.

[11] Fall M, Awbeck Fall A, Léye A, Ndiaye M, Moreira Diop T (2015). La myasthénie auto-immune de l'adulte lors d'une consultation décentralisée de neurologie au centre hospitalier national de Pikine dans la banlieue de Dakar-Sénégal. Rev Neurol 171.

[12] Gilhus NE, Nacu A, Andersen JB, Owe JF (2015). Myasthenia gravis and risks for comorbidity. Eur J Neurol.

[13] Gilhus NE, Verschuuren JJ (2015). Myasthenia gravis: subgroup classification and therapeutic strategies. Lancet Neurol.

[14] Gnonlonfoun D, Adjien KC, Mapaga JN, Goudjinou G, Mounguengui MM, Mouangue G, Houinato DS (2018). Myasthenie auto-immune: diagnostic et prise en charge. A propos de six cas au Benin et au Gabon. African Journal of Neurological Sciences.

[15] Grob D, Brunner N, Namba T, Pagala M (2008). Lifetime course of myasthenia gravis. Muscle Nerve.

[16] Heckmann JM, Owen EP, Little F (2007). Myasthenia gravis in South Africans: racial differences in clinical manifestations. Neuromuscul Disord.

[17] Huda S, Woodhall MR, Vincent A, Heckmann JM (2016). Characteristics Of acetylcholine-receptor-antibody-negative myasthenia gravis in a South African cohort. Muscle Nerve.

[18] Jaretzki A, Barohn RJ, Ernstoff RM, Kaminski HJ, Keesey JC, Penn AS, Sanders DB (2000). Myasthenia gravis: recommendations for clinical research standards. Task Force of the Medical Scientific Advisory Board of the Myasthenia Gravis Foundation of America. Neurology.

[19] Kawaguchi N, Kuwabara S, Nemoto Y, Fukutake T, Satomura Y, Arimura K, Osame M, Hattori T (2004). Study Group for Myasthenia Gravis in Japan. Treatment and outcome of myasthenia gravis: retrospective multi-center analysis of 470 Japanese patients, 1999-2000. J Neurol Sci.

[20] Keesey JC (2004). Clinical evaluation and management of myasthenia gravis. Muscle Nerve.

[21] Lompo DL, Cisse K, Yameogo MA, Napon C, Kabore BJ (2017). Myasthenia gravis at Ouagadougou (Burkina Faso): about 14 cases. Brain Nerves.

[22] Maggi L, Andreetta F, Antozzi C, Baggi F, Bernasconi P, Cavalcante P, Cornelio F, Muscolino G, Novellino L, Mantegazza R (2008). Thymoma-associated myasthenia gravis: outcome, clinical and pathological correlations in 197 patients on a 20-year experience. J Neuroimmunol.

[23] Maharaj J, Bahadursingh S, Ramcharan K (2013). Myasthenia gravis in South Trinidad. West Indian Med J..

[24] Mantegazza R, Baggi F, Antozzi C, Confalonieri P, Morandi L, Bernasconi P, Andreetta F, Simoncini O, Campanella A, Beghi E, Cornelio F (2003). Myasthenia gravis (MG): epidemiological data and prognostic factors. Ann N Y Acad Sci.

[25] Matsui N, Nakane S, Nakagawa Y, Kondo K, Mitsui T, Matsumoto T, Arisawa K, Kaji R (2009). Increasing incidence of elderly onset patients with myasthenia gravis in a local area of Japan. J Neurol Neurosurg Psychiatry.

[26] Matuja WB, Aris EA, Gabone J, mgaya EM (2001). Incidence and characteristics of Myasthenia gravis in Dar Es Salaam, Tanzania. East Afr Med J..

[27] Murai H, Yamashita N, Watanabe M, Nomura Y, Motomura M, Yoshikawa H, Nakamura Y, Kawaguchi N, Onodera H, Araga S, Isobe N, Nagai M, Kira J (2011). Characteristics of myasthenia gravis according to onset-age: Japanese nationwide survey. J Neurol Sci.

[28] Oh SJ, Morgan MB, Lu L, Hatanaka Y, Hemmi S, Young A, Claussen GC (2009). Racial differences in myasthenia gravis in Alabama. Muscle Nerve.

[29] Ojini FI, Danesi MA, Ogun SA (2004). Clinical manifestations of myasthenia gravis - review of cases seen at the Lagos University Teaching Hospital. Niger Postgrad Med. J.

[30] Onyekwulu FA, Onwuekwe IO (2010). Critical care of myasthenia gravis in a resource poor setting: a study of South East Nigeria. Neurologist.

[31] Peragallo JH, Bitrian E, Kupersmith MJ, Zimprich F, Whittaker TJ, Lee MS, Bruce BB (2016). Relationship Between Age, Gender, and Race in Patients Presenting With Myasthenia Gravis With Only Ocular Manifestations. J Neuroophthalmol.

[32] Sadri Y, Haghi-Ashtiani B, Zamani B, Akhundi FH (2015). Study of demographic, clinical, laboratory and electromyographic symptoms in Myasthenia Gravis patients referred to the neurology clinic of Rasoul Akram hospital in 2015. J Med Life.

[33] Sagna SD, Ndiaye M, Diop MS (2019). La myasthénie auto-immune de l'enfant: à propos de 20 cas colligés au centre hospitalo-universitaire de Dakar. Revue Neurologique.

[34] Yacouba K, Salaheddine M, Daniel AK, Christelle AC, Marcellin B, Maouly F, Amadou DG (2020). Therapeutic Itinerary of the Patients Followed for Myasthenia Gravis in Dakar. Clinical Neurology and Neuroscience.

[35] Zhang X, Yang M, Xu J, Zhang M, Lang B, Wang W, Vincent A (2007). Clinical and serological study of myasthenia gravis in HuBei Province, China. J Neurol Neurosurg Psychiatry.

